# Structural Communication between the *E. coli* Chaperones DnaK and Hsp90

**DOI:** 10.3390/ijms22042200

**Published:** 2021-02-23

**Authors:** Matthew P. Grindle, Ben Carter, John Paul Alao, Katherine Connors, Riina Tehver, Andrea N. Kravats

**Affiliations:** 1 Department of Chemistry & Biochemistry, Miami University, Oxford, OH 45056, USA; grindlm@miamioh.edu (M.P.G.); alaooj@miamioh.edu (J.P.A.); connorskg@miamioh.edu (K.C.); 2 Department of Physics, Denison University, Granville, OH 43023, USA; carter_b1@denison.edu (B.C.); tehverr@denison.edu (R.T.)

**Keywords:** chaperone, normal mode analysis, elastic network model, structural perturbation method, Hsp90, Hsp70, HtpG, DnaK, *E. Coli*

## Abstract

The 70 kDa and 90 kDa heat shock proteins Hsp70 and Hsp90 are two abundant and highly conserved ATP-dependent molecular chaperones that participate in the maintenance of cellular homeostasis. In *Escherichia coli*, Hsp90 (Hsp90${}_{Ec}$) and Hsp70 (DnaK) directly interact and collaborate in protein remodeling. Previous work has produced a model of the direct interaction of both chaperones. The locations of the residues involved have been confirmed and the model has been validated. In this study, we investigate the allosteric communication between Hsp90${}_{Ec}$ and DnaK and how the chaperones couple their conformational cycles. Using elastic network models (ENM), normal mode analysis (NMA), and a structural perturbation method (SPM) of asymmetric and symmetric DnaK-Hsp90${}_{Ec}$, we extract biologically relevant vibrations and identify residues involved in allosteric signaling. When one DnaK is bound, the dominant normal modes favor biological motions that orient a substrate protein bound to DnaK within the substrate/client binding site of Hsp90${}_{Ec}$ and release the substrate from the DnaK substrate binding domain. The presence of one DnaK molecule stabilizes the entire Hsp90${}_{Ec}$ protomer to which it is bound. Conversely, the symmetric model of DnaK binding results in steric clashes of DnaK molecules and suggests that the Hsp90${}_{Ec}$ and DnaK chaperone cycles operate independently. Together, this data supports an asymmetric binding of DnaK to Hsp90${}_{Ec}$.

## 1. Introduction

Molecular chaperones play an important role in maintaining homeostasis within the cell by participating in processes such as protein folding, protein remodeling, prevention of aggregation, and disaggregation [1,2,3,4,5,6]. Two highly abundant and evolutionary conserved chaperones include Heat Shock Protein 90 (Hsp90) and Heat Shock Protein 70 (Hsp70) [7,8,9,10,11,12]. They are present from bacteria to man and paralogs exist in multiple cellular locations. During cellular stress conditions, both chaperones are upregulated and overexpressed. Hsp90 and Hsp70 often collaborate in protein remodeling and activation of substrate proteins, termed “clients”, [13,14,15] including many regulatory proteins such as kinases, steroid hormone receptors, and transcription factors [9,12,16,17,18,19,20,21,22,23,24].

Members of the Hsp90 family of proteins assemble as homodimers with each protomer containing three separate domains: an N-terminal domain (NTD) that binds and hydrolyzes ATP, a middle domain (MD) that is the main locus of client binding and maturation, and a C-terminal domain (CTD) that is responsible for dimerization and client binding. ATP binding and hydrolysis by Hsp90 is essential for the binding, maturation, and release of client proteins [9,25,26,27]. In the absence of nucleotide, Hsp90 adopts an open “V”-shaped conformation, which can vary from extended to more compact in structure [28,29,30]. Nucleotide binding and hydrolysis induce large scale conformational changes that shift the structure to more closed conformations involving dimerization at both the N- and C-domains [30]. These ATP-dependent conformational changes are fundamental to the chaperoning mechanism and are conserved across species [27,31]; however, the dynamic equilibrium of occupancy in the various conformations differs [27,28,30,32,33,34,35]. In bacteria, Hsp90 is not essential though it displays modest growth defects at elevated temperature, subtle differences in growth phenotypes [36,37], and exhibits a small accumulation of protein aggregates [38]. In eukaryotes, Hsp90 is essential and remodels more than 300 client proteins. Although Hsp90 holds a key role in protein quality control in healthy cells, Hsp90 is a central protein in the propagation of cancer, as it has been shown to chaperone oncoproteins. Eukaryotic Hsp90 also has at least 20 cochaperones which modulate its ATPase activity and bias the conformational dynamics of Hsp90 to stabilize individual conformational states [39,40,41,42,43,44]. In contrast, bacterial Hsp90 does not have any identified cochaperones that participate in protein remodeling.

The Hsp70 family of molecular chaperones are also highly abundant and conserved. They are composed of an N-terminal nucleotide binding domain (NBD) and a C-terminal substrate binding domain (SBD), which are connected by a flexible linker that acts as a conformational switch to enable interdomain communication [45,46,47]. Substrate binding occurs between the β sandwich core and the α helical lid of the SBD (SBD-β and SBD-α, respectively) and differs in affinity based on the nucleotide bound state [10,48,49]. When ATP is bound, Hsp70 populates an “open” state that weakly binds substrate [50]. In this conformation, the SBD is docked onto the NBD. Conformational changes are induced by ATP hydrolysis that undocks the two domains and results in a high affinity for substrate within the SBD [46]. The Hsp70 chaperone cycle is facilitated by two cochaperones, J-domain proteins (Hsp40s) and nucleotide exchange factors (NEF) [51,52,53]. J-domain proteins stimulate ATP hydrolysis by Hsp70 [54,55], and NEF promote nucleotide exchange [56,57,58,59].

**Figure 1:**
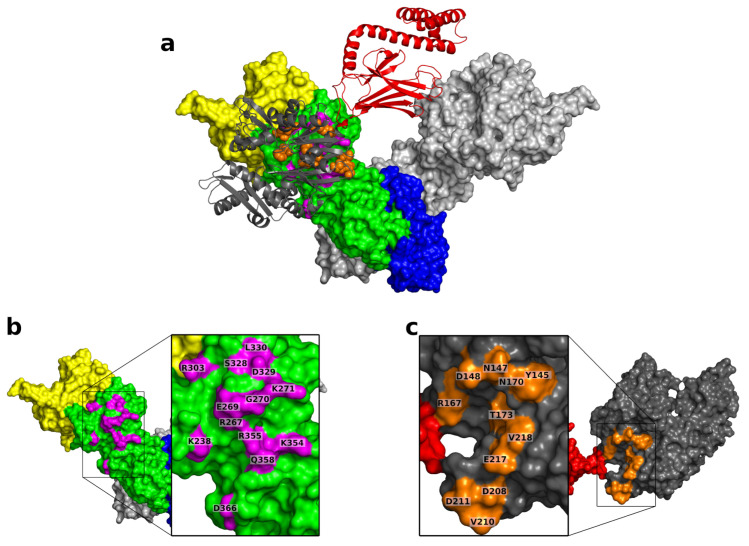
DnaK-Hsp90${}_{Ec}$ docked model. The residues of DnaK and Hsp90${}_{Ec}$ that are involved in the direct interaction have previously been described [65,66,72]. (**a**) Hsp90${}_{Ec}$-DnaK docked model. (**b**) Residues of Hsp90${}_{Ec}$ (magenta) that interact with DnaK are localized in the MD (green) outside of the cleft of the Hsp90${}_{Ec}$ dimer. (**c**) Residues of DnaK (orange) that interact with Hsp90${}_{Ec}$ are located in the NBD (grey).

Members of the Hsp70 and Hsp90 chaperone families collaborate in protein remodeling. Hsp70s act early in the protein folding pathway on proteins that are relatively unstructured [60], while Hsp90s work later in the folding pathway on more structured proteins [12,15,61,62,63]. In bacteria, the Hsp90 and Hsp70 (referred to as Hsp90${}_{Ec}$ and DnaK) chaperone systems collaborate synergistically to complete remodeling of a client protein [15,64]. This involves a direct interaction between Hsp90${}_{Ec}$ and DnaK in absence of cochaperones [64,65,66,67,68,69]. A genetic screen in *E. coli* identified a region located in the middle domain of Hsp90${}_{Ec}$ that was involved in the interaction with DnaK [65]. Furthermore, molecular docking studies identified a region on the nucleotide binding domain of DnaK that potentially interacted with Hsp90${}_{Ec}$ [66]. The predicted interaction is shown in (Figure 1). This region on DnaK was confirmed to be important for the interaction with Hsp90${}_{Ec}$ as substitution mutants in this region were defective in interaction with Hsp90${}_{Ec}$ in vitro [66]. Chemical cross-linking experiments further showed the interaction of the NBD of DnaK and the middle domain of Hsp90${}_{Ec}$ was direct and not the result of conformational changes elsewhere on the proteins, thus validating this computational model [70]. The bacterial chaperone systems have provided a useful tool in exploring the collaboration and direct interaction between the chaperones without additional complications from participation of other cochaperones throughout the chaperone cycle. There is also an abundance of structural information involving conformations in various nucleotide bound states [30,66], as well as functional information about the Hsp90-DnaK collaboration [12,15,64,66,67,70,71,72]. The direct interaction between Hsp70 and Hsp90 is conserved in yeast [67], suggesting potential similarities in mechanisms. However, the conformational dynamics and progression through the chaperone cycles are more complex and involve cochaperones [9,73]. For instance, the Hsp90-Hsp70 interaction can be bridged by the Hop/Sti1 chaperone that interacts simultaneously with both chaperones and stabilizes the open conformation of Hsp90 by inhibiting Hsp90 ATPase activity [7,13,14,39,74,75,76,77]. Given the sequence and structural homology of the chaperones and the conserved direct interaction in yeast, mechanistic details in the bacterial system may also be conserved in higher eukaryotes.

The coordination of the Hsp90 and Hsp70 ATPase cycles and how the allosteric signal is transmitted throughout this complex to regulate the ATP-dependent conformational changes of both chaperones remains unclear. In this work, we set out to understand how Hsp90${}_{Ec}$ and DnaK allosterically communicate and couple their ATP-dependent conformational cycles. The large system size and the conformational changes of both chaperones make molecular dynamics simulations challenging. A simplified mathematical approach is to consider the dominant vibrational modes using network models. Elastic network models have been successful in studying large scale motions of other biological systems and long distance communications between conformational states [78,79,80,81,82,83,84,85,86,87,88,89,90]. Normal mode analysis of these fluctuations reveal the low frequency modes corresponding to large scale conformational changes [91], which have been reported to resemble dynamics obtained from more accurate simulations [92,93]. In this study, we use an elastic network model (ENM) and normal mode analysis (NMA) to study the conformational changes of Hsp90${}_{Ec}$. We then probe the effects of a bound cochaperone, DnaK, and the stoichiometry between Hsp90${}_{Ec}$ and DnaK by using asymmetric and symmetric DnaK bound structures. We also use the Structural Perturbation Method (SPM) to identify amino acids responsible for transmitting the allosteric signals [85,94].

## 2. Materials and Methods

Using the low frequency modes predicted by ENM has been shown to successfully describe global motions of proteins and complexes, including structural transitions that connect two allosteric states [85,88,95,96,97,98]. We have modeled all proteins as elastic networks composed of *N* nodes where *N* is the number of amino acids in the PDB structures [95]. Each node is located at the α-carbon atom of an amino acid residue; the nodes that are within a cutoff distance $R_{c}=9$ Å in the PDB structure (see Preparation of Structures), are connected via harmonic potential with the energy function:

(1) $$H=\frac{1}{2}\sum_{i,j:d_{ij}^{0}<R_{c}}\gamma {\left(d_{ij}-d_{ij}^{0}\right)}^{2}$$

where γ is the spring constant that defines the energy scale, $d_{ij}$ is the dynamic distance between residues *i* and *j*, and $d_{ij}^{0}$ is the corresponding PDB distance. The dynamics of the system is obtained by calculating the normal modes of the mass-spring system with potential energies given by Equation (1). The normal mode calculation yields a set of 3N-dimensional eigenvectors, $q_{M}$ and corresponding eigenvalues $\omega_{M}$ for each mode *M*. The cutoff distance ($R_{c}=9$ Å) was chosen using a comparison of the normal mode-based calculated B-factors to the B-factors reported in PDB structures, where available [89]. The same spring constant was used in all three models.

### 2.1. Measured Quantities

The overlap function is used to describe how closely a single mode matches the allosteric change of dynamic proteins with multiple conformational states. The calculation for the structural transition is given by:

(2) $$I_{M}=\frac{\sum q_{iM}\Delta r_{i}}{\sqrt{\sum q_{iM}^{2}}\sqrt{\sum \Delta r_{i}^{2}}}$$

where *M* is the mode index, $\Delta r_{i}$ is the difference in the locations of the *i*th amino acid α-carbon atom in the two structures that correspond to the starting and end conformations of a structure, and $q_{iM}$ are the corresponding eigenvectors. The sums are over all nodes and thus include 3N terms. Based on the definition in Equation (2), $0\leq I_{M}\leq 1$. The closer the value is to 1, the more accurately a given mode describes the structural transition between the two states.

To quantify the pairwise correlations of amino acid vibrations and highlight domain motions, we calculate the covariance matrix as:

(3) $$C_{ij}=\frac{\sum q_{iM}q_{jM}/\omega_{M}}{\sqrt{\sum q_{iM}^{2}/\omega_{M}}\sqrt{\sum q_{jM}^{2}/\omega_{M}}}$$

where the sums are over the modes, *M*. Since $-1\leq C_{ij}\leq 1$, the regions where $C_{ij}$ values are close to +1 correspond to concerted vibrations, negative $C_{ij}$ values indicate motions that are in opposite directions.

The relative displacement of a node *i* in mode *M* is calculated from the normalized eigenvectors $q_{iM}$ as:

(4) $$\delta q_{iM}=\sqrt{q_{i_{x}M}^{2}+q_{i_{y}M}^{2}+q_{i_{z}M}^{2}}$$

where $q_{i_{u}M}$ denotes the displacement of the site *i* in the *u* direction.

#### 2.1.1. Structural Perturbation Method (SPM)

The structural perturbation method (SPM) was developed to assess the dynamic role of individual amino acids in a structural transition [86,99]. The SPM allows us to quantify how a mutation of an amino acid alters the allosteric dynamics of an entire protein or protein complex. In practice we calculate the response to a mutation at the site *i* as a perturbation:

(5) $$\delta \omega_{iM}=\frac{1}{2}\sum_{j:d_{ij}^{0}<R_{c}}\delta \gamma {\left(d_{ij}-d_{ij}^{0}\right)}^{2}$$

where δγ is the perturbed spring constant. It is important to note that while the sum only includes amino acids within the cutoff $R_{c}$ of the mutation site, the resulting changes in the eigenvectors encompass the entire elastic network. Thus, the greater the response $\delta \omega_{iM}$, the more dynamically significant a specific residue is to a given mode. In other words, high $\delta \omega_{iM}$ nodes trace a network of residues that can be considered an allostery wiring diagram for a transition. We highlight the nodes that have a top 2% of $\delta \omega_{iM}$ values. To determine whether hot-spot residues were conserved across the Hsp90 and Hsp70 families of chaperones, Hsp90${}_{Ec}$ and DnaK amino acid sequences were aligned to relevant family member sequences using Clustal Omega [100]. Amino acid sequences were collected from Uniprot [101].

#### 2.1.2. Preparation of Structures

The structures were prepared as described previously [66]. Briefly, missing atoms of the ADP bound conformation of DnaK (PDB ID 2KHO [46]) were built in using the CHARMM molecular modeling program [102]. The Hsp90${}_{Ec}$ dimer in the apo conformation was obtained from PDB ID 2IOQ (biological assembly 1) [30]. Missing regions of a single protomer were modeled using ITASSER [103,104,105] and then two identical models of each protomer were overlayed with the biological assembly to produce a full length homodimer. The proteins were docked using ZDOCK [106,107]; the top 2000 complexes were reranked using ZRANK [108]; and the highest scoring complex was taken. To create the symmetric complex, we started with the highest scoring docked model. A second Hsp90${}_{Ec}$ monomer bound to DnaK was overlayed with the Hsp90${}_{Ec}$-DnaK complex as to minimize the root mean square deviation between the bound and Hsp90${}_{Ec}$ protomers. The corresponding DnaK coordinates were added to the Hsp90${}_{Ec}$-DnaK complex to create a symmetric complex. The Hsp90${}_{Ec}$ alone and Hsp90${}_{Ec}$-DnaK complexes were then reduced from their all-atom representation to carbon-α only configuration for subsequent ENM calculations [78,79,80,81,82,83,95]. The structural overlap was computed using the ADP bound conformation of Hsp90${}_{Ec}$, PDB ID 2IOP [30]. This structure was modeled using the same procedure as the apo conformation, described above, to ensure the same number of atoms for reference.

## 3. Results

### 3.1. The Conformational Transition of Hsp90${}_{Ec}$ from the Open → Close Conformation Can Be Described by Multiple Normal Modes

**Figure 2:**
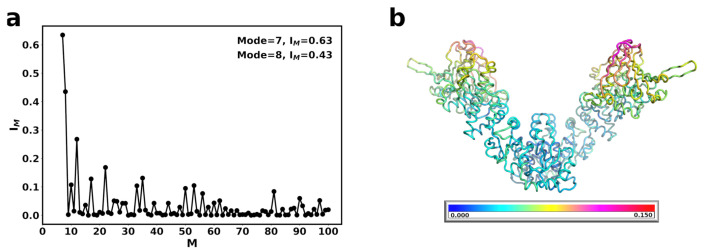
Normal modes of apo Hsp90${}_{Ec}$ (**a**) Structural overlap of the normal modes of apo Hsp90${}_{Ec}$ with ADP bound Hsp90${}_{Ec}$ to identify the modes that contribute to the biological movement. The closed circles represent the overlap for individual modes. Lines are drawn for clarity. Nonzero modes with the highest structural overlap are considered. (**b**) The sum of the individual amino acid fluctuations in both modes 7 and 8 are represented on the structure of Hsp90${}_{Ec}$. Blue represents static residues while red highlights highly mobile residues.

**Figure 3:**
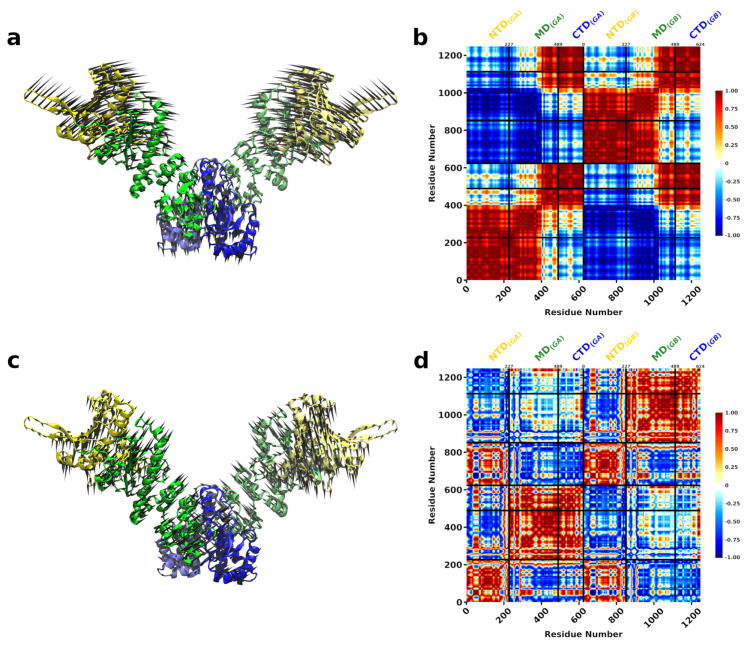
Motions associated with significant normal modes of Hsp90${}_{Ec}$ alone. (**a**) Mode 7 consists of swing motions of each protomer that contributes to closing and dimerization. (**b**) Covariance matrix of amino acid pairs for Mode 7 (**c**) Mode 8 consists of torsional motions about the CTD. (**d**) Covariance matrix of amino acid pairs for modes 8. Arrows in (**a**,**c**) indicate the amplitude and direction of motions of amino acids in each mode. The color scheme highlights the Hsp90${}_{Ec}$ N-domain in yellow, M-domain in green, C-domain in blue. Correlated movements in covariance matricies (**b**,**d**) are highlighted in red while anticorrelated movements are represented in blue.

The normal modes of Hsp90${}_{Ec}$ in the apo conformation were calculated and compared against the ADP bound state of Hsp90${}_{Ec}$, since these modes are considered to contribute to the biological motion of the protein. The contribution of individual normal modes with significant structural overlap values ≥0.35 were considered (Figure 2a, Methods). The movement of Hsp90${}_{Ec}$ from the apo to the ADP bound conformation is best described by two dominant modes. In the first mode, Mode 7 (0.63 overlap), the dimer undergoes a scissoring motion with both protomers moving towards each other (Figure 3a and Supplementary Movies S1 and S2). To further understand the movement, the collective motions of the amino acids are quantified by the correlated motion of pairs of amino acids in each individual mode, where positive correlations (red) indicate amino acids are moving in the same direction, negative correlations (blue) indicate movement in opposite directions, or no correlation is observed (white). Figure 3b shows the correlation matrix for Mode 7 of Hsp90${}_{Ec}$, where one protomer of Hsp90${}_{Ec}$ consists of residues 1 to 624 and the second protomer consists of residues 625 to 1248. Mode 7 is characterized by correlated motions in the N-M and the M-C domain; the NTD and half of the MD move as a rigid body, and the CTD and the other half of the MD move as a rigid body. The N-M and M-C motions are anticorrelated within a protomer (Figure 3a,c). Additionally, the N-M motions are anticorrelated between both protomers due to the swinging inward motion that results in the transition from the open “V” structure to the closed structure dimerized at the the C and N domains. The M-C motions are correlated between protomers with this region on both protomers swinging downward to accommodate the closing of the protomers.

The second dominant mode, Mode 8 (0.44 overlap), involves a torsional motion about the CTD domain (Figure 3c, Supplementary Movies S3 and S4). In this mode, the NTD and the M-C domains move as rigid bodies (Figure 3d). The motions of the NTD of both protomers are correlated, while the M-C motions across protomers are anticorrelated due to a torsion involving closing/opening of the structure. The cross-correlation matrices for the dominant modes are consistent with principle component analysis obtained from molecular dynamics simulations of Hsp90${}_{Ec}$ in the apo conformation [109]. We further quantified the magnitude of the motions of each individual amino acid for each mode, $\delta q_{iM}$ (Methods). These values can be considered similar to temperature factors [97] and describe relative displacements within each mode. The sum of these movements in both dominant modes were mapped onto the structure of Hsp90${}_{Ec}$ (Figure 2b) and shown individually for each mode in Supplementary Figure S1. The coloring on the structure begins with no mobility (dark blue) and continues through the spectrum to high mobility (red). The residue fluctuations for the dominant modes of Hsp90${}_{Ec}$ indicate rigidity in the CTD and a portion of the MD, involving some of the experimentally identified residues in the DnaK binding region [66]. The highest mobility is observed in the NTD and a region of the MD. The lid of the ATP binding pocket (residues 109–118) exhibits limited mobility in both protomers with relative $\delta q_{iM}$ values for individual modes ≃0.03 (Supplementary Figure S1). Overall, in both dominant modes, Hsp90${}_{Ec}$ acts symmetrically with both protomers displaying similar behavior in the covariance matrices (Figure 3) and displacements (Figure 2b and Supplementary Figure S1) with nearly identical patterns and fluctuations.

### 3.2. A Single DnaK Molecule Modulates the Conformational Flexibility of the Bound Hsp90${}_{Ec}$ Protomer

**Figure 4:**
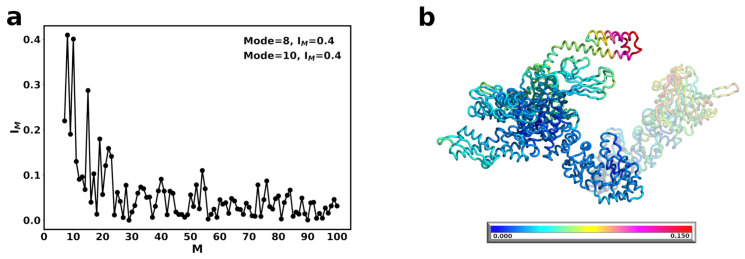
Normal modes of asymmetric DnaK bound Hsp90${}_{Ec}$ (**a**) Structural overlap of DnaK bound Hsp90${}_{Ec}$ model with ADP bound Hsp90${}_{Ec}$ to identify the dominant modes that contribute to the biological movement. The closed circles represent the overlap for individual modes. Lines are drawn for clarity. Nonzero modes with the highest structural overlap are considered. (**b**) The sum of the individual amino acid fluctuations in both modes 8 and 10 are represented on the structure of Hsp90${}_{Ec}$. Blue represents static residues while red highlights highly mobile residues.

**Figure 5:**
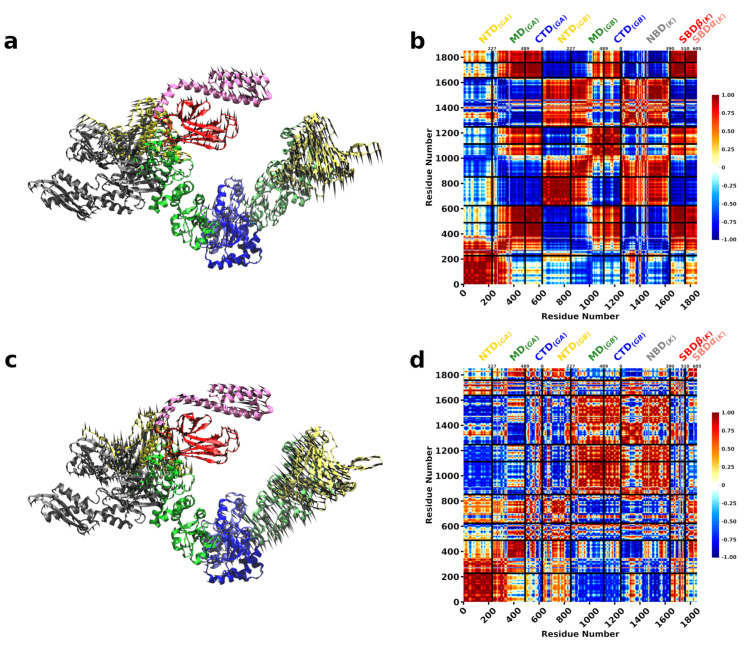
Motions associated with significant normal modes of Hsp90${}_{Ec}$ when one DnaK is bound. (**a**) Mode 8 involves a scissoring motion about the CTD domains of Hsp90${}_{Ec}$ with DnaK undergoing torsional motions perpendicular to the protomer of Hsp90${}_{Ec}$ which it is bound. The DnaK SBD is placed into the client binding site of Hsp90${}_{Ec}$ (**b**) Covariance matrix of amino acid pairs for Mode 8 (**c**) Mode 10 is a torsional motion of Hsp90${}_{Ec}$ about the CTD dimerization domain with DnaK undergoing torsions about the same axis as Hsp90${}_{Ec}$. (**d**) Covariance matrix of amino acid pairs for modes 10. The DnaK SBD-α and SBD-β open to potentially release a client. Arrows in (**a**,**c**) indicate the amplitude and direction of motions of amino acids in each mode. The NBD of DnaK is colored in grey with the SBD-α in mauve and the SBD-β in red. Correlated movements in covariance matricies (**b**,**d**) are highlighted in red while anticorrelated movements are represented in blue.

Next, we wanted to understand how the presence of DnaK affects the fluctuations of Hsp90${}_{Ec}$ in the open to close transition. For this calculation, we used the docked model of Hsp90${}_{Ec}$-DnaK [66] (Figure 1) that considered apo Hsp90${}_{Ec}$ and ADP-bound DnaK. Based on the structural overlap, two dominant modes were identified, modes 8 and 10, each accounting for about 40 percent of the conformational transition of Hsp90${}_{Ec}$ (Figure 4a). The first dominant mode, Mode 8, involves a scissoring motion about the CTDs of Hsp90${}_{Ec}$ (Figure 5a, Supplementary Movies S5 and S6). The covariance matrices are represented for the first protomer of Hsp90${}_{Ec}$ (bound to DnaK) consisting of residues 1 to 624, followed by the second protomer of Hsp90${}_{Ec}$ (unbound) consisting of residues 625 to 1248, while residues 1249 to 1853 correspond to DnaK (Figure 5b,d). In the covariance matrix for Mode 8 (Figure 5b), there are correlations about the N-M and M-C domains of individual protomers, as observed in the calculations of Hsp90${}_{Ec}$ alone (Figure 3). Additionally, DnaK acts as a rigid body with the NBD and SBD exhibiting high correlations within domains and anticorrelation between the two domains. Movement of the DnaK SBD is highly correlated with the movement of the M-C domain of which it is bound. This rotational motion orients the substrate binding domain of DnaK centered within the substrate binding site of Hsp90${}_{Ec}$ [71], poised to deliver a client protein. The NBD of DnaK moves anticorrelated to the region in the middle domain of which it is bound; instead it is highly correlated to the motion of N-M domain of the opposite unbound Hsp90${}_{Ec}$ protomer. The presence of DnaK bound to the first protomer of Hsp90${}_{Ec}$ changes the interdomain correlation relative to the unbound protomer; there is less correlation between the N-M-domain and a higher correlation in the M-C domain by comparison.

The second dominant mode, Mode 10, is characterized by a torsional motion about the CTD dimerization domain (Figure 5c and Supplementary Movies S7 and S8). In Mode 10, relatively high correlations are observed in N-M and M-domains are observed in the DnaK bound Hsp90${}_{Ec}$ protomer (Figure 5d), suggesting rigid body movements. In contrast, high correlations are observed in M-C domains in the unbound Hsp90${}_{Ec}$ protomer. The binding of DnaK to one Hsp90${}_{Ec}$ protomer removes the symmetry that was previously observed in the Hsp90${}_{Ec}$ alone model. Additionally, correlations are observed within the individual domains of DnaK indicating that the NBD, SBD-α and SBD-β all move as separate rigid bodies. There is a strong anticorrelation between the SBD-α and SBD-β. This is the region that a substrate protein would be bound in the DnaK ADP conformation. These movements indicate a possible release mechanism of a substrate protein from the DnaK SBD to the Hsp90${}_{Ec}$ substrate binding region [71].

The overall magnitude of relative amino acid displacements in both dominant modes follow a similar trend, with suppressed fluctuations in the Hsp90${}_{Ec}$ protomer that is bound to DnaK by an average of 2 fold in comparison to the unbound protomer (Supplementary Figure S2). Hsp90${}_{Ec}$ residues that are in contact with DnaK and residues in the M-C domain are relatively immobile (displacement ≤ 0.02). This results in a stabilization of the substrate binding residues of the DnaK bound Hsp90${}_{Ec}$ protomer, though not the unbound protomer, which still exhibits much more flexibility by comparison (Supplementary Figure S2). Figure 4b highlights the sum of the fluctuations in both modes, showing immobility of the bound Hsp90${}_{Ec}$ protomer and the DnaK NBD and conversely the flexibility of the opposite Hsp90${}_{Ec}$ protomer and the highly mobile region on the SBD of DnaK. Taken together, these motions could indicate a potential role in client protein hand-off. DnaK acts to stabilize the Hsp90${}_{Ec}$ protomer and prevent it from undergoing conformational changes similar to some cochaperones like Hop or Sti1 of eukaryotic Hsp90s. This increased stabilization encompasses the substrate binding region of Hsp90${}_{Ec}$, which provides a stable interacting surface for a substrate to bind. Similarly, residues in the DnaK NBD that are located in the ATP binding pocket (K70, P143, Y145, F146, R151, E171) or in contact with the Hsp90${}_{Ec}$ middle domain (Figure 1c) are immobile (relative displacement <0.02) and potentially act to stabilize the bound configuration of the chaperones to ensure client protein delivery. The residues of the ATP binding lid of Hsp90${}_{Ec}$ also exhibit limited mobility in both protomers relative to the Hsp90${}_{Ec}$ alone model, with relative displacements from ≃0.025 to 0.04 in either protomer when DnaK is bound. In contrast, the most mobile residues aside from those in the N domain of the unbound protomer are located in the α-helical portion of the SBD of DnaK, potentially acting to release a bound client protein from DnaK to Hsp90${}_{Ec}$. The fluctuations in both dominant modes of DnaK are in agreement with several other studies of Hsp70/DnaK that report rigid body movements of the NBD and SBD [110], large fluctuations within the SBD and small fluctuations in the NBD [111], and the opening between the SBD-α and SBD-β [112].

### 3.3. DnaK-Hsp90${}_{Ec}$ Stoichiometry of 1:1 Returns the Conformational Flexibility and Symmetry within Hsp90${}_{Ec}$

**Figure 6:**
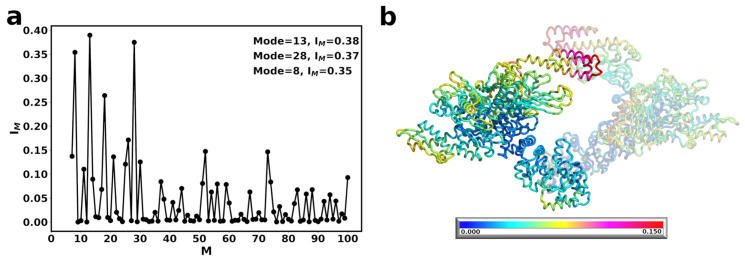
Normal modes of symmetric DnaK bound Hsp90${}_{Ec}$ (**a**) Structural overlap of the two DnaK bound Hsp90${}_{Ec}$ model with ADP bound Hsp90${}_{Ec}$ to identify the dominant modes that contribute to the biological movement. The closed circles represent the overlap for individual modes. Lines are drawn for clarity. Nonzero modes with the highest structural overlap are considered.(**b**) The sum of the individual amino acid fluctuations in both modes 13, 28, and 8 are represented on the structure of Hsp90${}_{Ec}$. Blue represents static residues while red highlights highly mobile residues.

**Figure 7:**
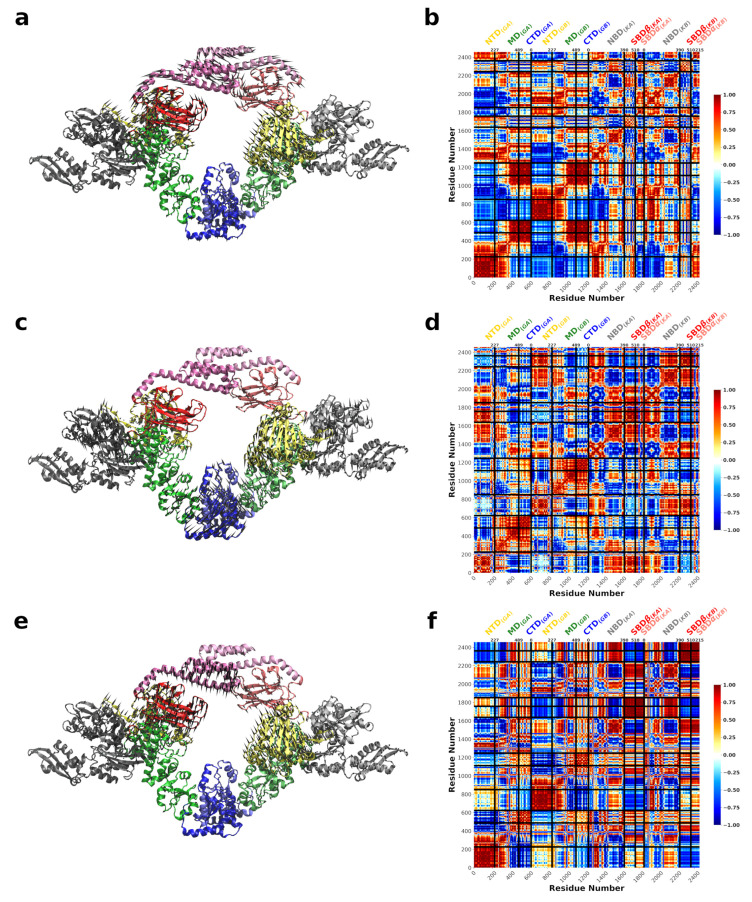
Motions associated with significant normal modes of one Hsp90${}_{Ec}$ dimer when two DnaK monomers are bound. (**a**) Mode 13 is characterized by a torsional motion about the C-terminus of Hsp90${}_{Ec}$. The DnaK SBDs are oriented toward the client binding region of Hsp90${}_{Ec}$ resulting in steric clashes. (**b**) The covariance matrix of amino acid pairs for Mode 13. (**c**) Mode 28 involves a torsional motion of Hsp90${}_{Ec}$ about the CTD dimerization domain. The NBD of both DnaK molecules moves away from the axis of Hsp90${}_{Ec}$ while the DnaK SBDs are immobile. (**d**) The covariance matrix of amino acid pairs for Mode 28. (**e**) Mode 8 also involves a torsional motion about the Hsp90${}_{Ec}$ CTD similar to Mode 13, but with the SBDs of each DnaK moving away from the Hsp90${}_{Ec}$ vertex. (**f**) Covariance matrix of amino acid pairs for Mode 8. Arrows in (**a**,**c**,**e**) indicate the amplitude and direction of motions of amino acids in each mode. Correlated movements in covariance matrices (**b**,**d**,**f**) are highlighted in red while anticorrelated movements are represented in blue.

Symmetry in Hsp90 complexes and remodeling mechanisms remains an open question in the field, with preference toward asymmetry [7,113,114,115,116,117]. In order to address symmetry for DnaK binding we produced a Hsp90${}_{Ec}$-DnaK model with a 1:1 stoichiometry. We modeled a second DnaK, also in the ADP bound conformation, onto the opposite Hsp90${}_{Ec}$ protomer (see Methods) and then carried out the ENM calculations and computed the vibrational modes. The functional overlap of these modes with the closed state of Hsp90${}_{Ec}$ (Figure 6a) reveals three dominant modes, modes 13, 28, and 8, that contribute about equally with 35–38% overlap with the eigenvectors to the closed state. The fluctuations of Hsp90${}_{Ec}$ in Mode 13 in the symmetric DnaK bound model are highly comparable to Mode 8 movements in the Hsp90${}_{Ec}$ conformation in the absence of DnaK, characterized by a torsional motion about the CTD of Hsp90${}_{Ec}$ (Figure 7a, Supplementary Movies S9 and S10). The covariance matrices are represented for the first protomer of Hsp90${}_{Ec}$ (bound to DnaK molecule 1) consisting of residues 1 to 624, followed by the second protomer of Hsp90${}_{Ec}$ (bound to DnaK molecule 2) consisting of residues 625 to 1248, while residues 1249 to 1853 correspond to DnaK molecule 1 and residues 1854 to 2458 correspond to DnaK molecule 2 (Figure 7b,d,f). There is a high correlation in movements within the N-M and M-C domains of Hsp90${}_{Ec}$ (Figure 7b); however, the fluctuations are suppressed about 2 fold relative to Mode 8 when DnaK was not bound (Supplementary Figure S1 (red), Supplementary Figure S3 (black)). In Mode 13, the DnaK molecules move anticorrelated with each other. Within a DnaK molecule there are high correlations within the NBD, SBD-α, and SBD β. The motions of the SBD-α and SBD-β are anticorrelated, which would result in opening of the SBD-α and SBD-β to potentially release a client protein from DnaK. Additionally, a movement of the DnaK SBD into the client binding site of Hsp90${}_{Ec}$ is observed and an opening of the SBD of DnaK molecules to potentially transfer a client protein to Hsp90${}_{Ec}$. However, the symmetric movements of the DnaK molecules in opposite directions would result in a clash of the DnaK SBD’s in the substrate binding region of Hsp90${}_{Ec}$, rendering this motion of low biological relevance.

The second dominant mode, Mode 28 reflects a torsional type motion of Hsp90${}_{Ec}$ with rotations of the CTD (Figure 7c, Supplementary Movies S11 and S12). The NTD and the M-C domains of Hsp90${}_{Ec}$ move as rigid bodies (Figure 7d) with both protomers moving symmetrically and both DnaK molecules moving symmetrically. In this mode the M-C domains of both protomers are moving together with high correlation, while the N-M domains of both protomers and both DnaK molecules are moving anticorrelated. There is also high correlation within individual DnaK molecules in the NBD and the SBD, where subdomains α and β move together with high correlation. The DnaK SBD and the Hsp90${}_{Ec}$ M-domain exhibit the smallest overall fluctuations in this mode (Supplementary Figure S3, red), which also include the sites of direct interaction on both proteins. The largest displacements are located on regions in the N- and C- domains of Hsp90${}_{Ec}$ protomers and the NBD of both DnaK molecules.

The third dominant mode, Mode 8, consists of a torsional motion of the Hsp90 protomers with DnaK moving in concert (Figure 7e, Supplementary Movies S13 and S14). The fluctuations in Mode 8 appear similar to those of Mode 13 with symmetric rigid-body movements of the Hsp90${}_{Ec}$ N-M and M-C domains (Figure 7b,f). However, the fluctuations of DnaK move outward from the Hsp90${}_{Ec}$ client binding pocket, rendering it incompatible with client transfer. In Mode 8, there is a very strong correlation of the entire SBD of both DnaK molecules. The fluctuations of individual amino acids in Mode 8 is also very similar to Mode 13 (Supplementary Figure S3, blue and black, respectively), with the largest displacements in the DnaK SBD and Hsp90${}_{Ec}$ NTD and CTD while the Hsp90Ec MD and DnaK NBDs are relatively immobile.

Taken together, the presence of the second DnaK molecule to create a symmetric complex with 1:1 stoichiometry returns the fluctuations of the system to symmetry. In all three modes, symmetric patterns of covariance were observed for the Hsp90${}_{Ec}$ protomers and both DnaK molecules. In addition, the relative fluctuations of the Hsp90${}_{Ec}$ residues in the symmetric DnaK complex are decreased (Figure 6b) compared to Hsp90${}_{Ec}$ alone (Figure 2b); this rigidity in Hsp90${}_{Ec}$ is symmetric. In contrast, the fluctuations in the symmetric DnaK model suggest this complex is less rigid than the asymmentric DnaK model (Figure 4b), that only suppressed the Hsp90${}_{Ec}$ fluctuations in the DnaK bound protomer. Furthermore, the DnaK molecules are more mobile in their NBD, linker, and SBD-β regions in the symmetric model, while these regions are rigidified in the asymmetric model. Figure 6b highlights the fluctuations of individual amino acids in the symmetric complex, summed over the three dominant modes. Similar to what was observed in the asymmetric DnaK model, the ATP binding lid for Hsp90${}_{Ec}$ exhibits limited mobility, with relative fluctuations between ≃0.05 and 0.07. Similarly, the residues of DnaK located in the ATP binding pocket are also relatively immobile with displacements ≤0.025.

### 3.4. SPM Analysis Reveals a Change in the Allosteric Wiring Network When DnaK Is Bound

**Figure 8:**
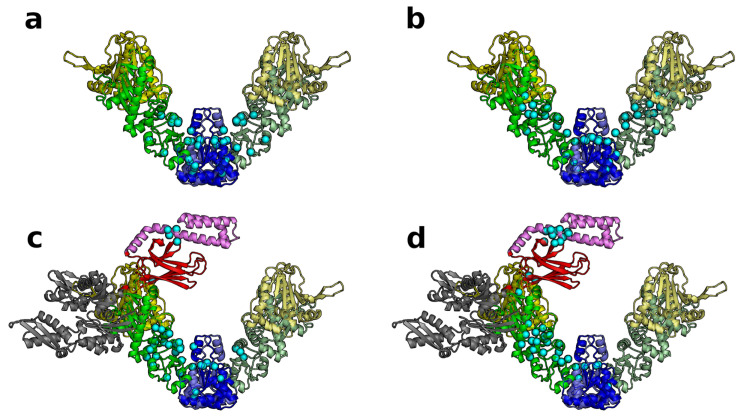
Hot-spot residues for the Hsp90${}_{Ec}$ and asymmetric Hsp90${}_{Ec}$-DnaK models. The top 2% (38) residues were mapped on the structures as cyan beads to indicate the amino acids involved in the allosteric wiring diagrams for each mode. (**a**,**b**) unbound Hsp90${}_{Ec}$ modes 7 and 8 (**c**,**d**) asymmetric Hsp90${}_{Ec}$-DnaK modes 8 and 10, respectively. All two-dimensional graphs in this paper including displacement graphs and correlation matrices were made using Python [122] and MatPlotLib [123]. Three-dimensional protein models were made with both PyMol [124] and VMD [125]. Raster images were edited using GIMP [126] and vector images were edited using Inkscape [127].

The structural perturbation method (SPM) identifies residues that are critical for function (see Methods). These can be considered “hot-spot” residues responsible for transmitting the allosteric signal for the large scale conformational changes. We focus on the top ≃2% (38) of residues involved in this network for each of the models. The residues involved in the allosteric wiring for the two dominant modes of the Hsp90${}_{Ec}$ alone model are shown as cyan spheres in Figure 8a,b and listed separately in Supplementary Table S1. The number of conserved residues in Supplementary Table S1 indicate that about two thirds of the residues involved in allosteric wiring in each model are conserved. In the Hsp90${}_{Ec}$ alone model, hot-spot residues are symmetric in both protomers and involve residues in the CTD and M-domain of Hsp90${}_{Ec}$. This allosteric network involves one residue determined to be important for substrate binding (E466) [71]. In contrast, the the hot-spot residues of Hsp90${}_{Ec}$ in the asymmetric DnaK bound model are distributed asymmetrically within each protomer (Figure 8c,d) for both dominant modes. Overall, many of the same Hsp90${}_{Ec}$ residues involved in the allosteric signal transmission are shared with the Hsp90${}_{Ec}$ alone model (highlighted in blue in Supplementary Table S1). The residues involved in the allosteric wiring of the DnaK-bound protomer now incorporate two additional residues involved in the client binding site (W467 and L553, in addition to E466). These substrate binding residues were shown to be defective in ATPase activity and in protein remodeling assays with the DnaK chaperone system [71], which is supported by their importance in transmitting allosteric signals. This SPM data for Hsp90${}_{Ec}$ is in agreement with the normal mode observations (Figure 4 and Figure 5) that indicate asymmetry, as the Hsp90${}_{Ec}$ protomer with DnaK is rigidified and does not move similarly to the unbound protomer. The allosteric signaling network DnaK bound Hsp90${}_{Ec}$ protomer involves additional residues in the N- and M-domains, some of which are near but not directly involved in the interaction with DnaK. Some DnaK residues involved in this network are located within the flexible linker, which is responsible for transmitting the allosteric signal between the NBD and the SBD. The SBD residues are located at the SBD-α-SBD-β interface that is involved in opening to allow substrate binding or release. Similar residues have been reported to be involved in the allosteric signaling of DnaK [118,119,120]. This SPM data further supports the hand-off of client from DnaK to Hsp90${}_{Ec}$ given the involved residues in the SBD of DnaK and the propagation of allostery from the C-domain to the region where DnaK is bound on Hsp90${}_{Ec}$.

While the symmetric DnaK bound Hsp90${}_{Ec}$ model does not seem biologically relevant due to the steric clashes in one of the dominant modes, we also performed the SPM analysis on this model. Interestingly, the hot-spot residues predominantly involved either DnaK or Hsp90${}_{Ec}$ (Supplementary Figure S4, Supplementary Table S1). These results suggest that in these modes, DnaK and Hsp90${}_{Ec}$ are working independently of each other. This would be counterproductive in contrast to a model where the allosteric signal is communicated throughout the bound complex to coordinate the Hsp90${}_{Ec}$ and DnaK ATP-dependent chaperone cycles. The hot-spot residues of DnaK and Hsp90${}_{Ec}$ are located in residues involved in the direct interaction [66]. This region on the NBD of DnaK is important for the interaction with the SBD of DnaK as well as cochaperones in the J-protein family. Additionally, residues of DnaK including R272, R261, Y15, Y41, K71, R72, E175, and H227 have been shown to be important for allosteric communication in the Hsp70 ATPase domain [121]. However, in these models none of these residues are utilized in any of the dominant modes, suggesting a rewiring of the allosteric mechanism when interacting with Hsp90${}_{Ec}$.

## 4. Discussion

This work explores the coupling of the Hsp90${}_{Ec}$ and DnaK chaperone cycles by their direct interaction. We probe the fluctuations of three different Hsp90${}_{Ec}$ models with 0, 1, and 2 DnaK molecules bound to dissect the role of symmetry in binding. The covariance and displacement plots indicate a high symmetry of motions in the models without DnaK or with symmetrical DnaK binding. When one DnaK is bound to Hsp90${}_{Ec}$, the fluctuations are suppressed in the Hsp90${}_{Ec}$ protomer that is bound to DnaK. This could act to stabilize the apo conformation of that protomer and prevent conformational changes to the closed state. ENM calculations of other Hsp90-cochaperone complexes [128,129] reveal some similarities to DnaK binding. For instance the kinase client recruiter, Cdc37, binding to Hsp90 causes the mobile ATP binding lid of Hsp90 to become rigid. Binding of the cochaperone Aha1 causes long range perturbations in the structure across multiple domains and increases stability in these regions. The late acting cochaperone p23 increases structural rigidity throughout residues in the N-, M-, and C- domains of Hsp90 as well. While these studies were performed with yeast or human Hsp90 structures, the homologous residues impacted in Hsp90${}_{Ec}$ differ from identified hot-spot residues in our studies, suggesting that DnaK is acting by a distinct mechanism.

Fluctuations within the asymmetrical model of Hsp90${}_{Ec}$ with one DnaK molecule appear to have biological relevance, with the placement of the DnaK SBD over the client binding site of Hsp90${}_{Ec}$ and the shearing of SBD-α and SBD-β of DnaK to release a client protein. SPM analysis of this model reveals a similar wiring in Hsp90${}_{Ec}$ to the Hsp90${}_{Ec}$ alone model, with additional hot-spots identified in the client binding region of Hsp90${}_{Ec}$ and in the SBD and interdomain linker of DnaK. In contrast, the symmetric DnaK bound model produces fluctuations that result in steric clashes of the DnaK SBDs. These fluctuations result in a less stable protein-protein interface. Only the regions of Hsp90${}_{Ec}$ where DnaK is bound are stabilized, as opposed to the entire DnaK bound protomer in the asymmetric model. Additionally, the DnaK NBD, linker, and SBD-β are more mobile than in the asymmetric model. The SPM analysis of the symmetric model revealed that the chaperone cycles were not coupled in two of the three dominant modes, with the majority of hot-spot residues located in either Hsp90${}_{Ec}$ or DnaK. Taken together, this data supports a model of asymmetric DnaK binding.

Mechanistic asymmetry in Hsp90 remains a highly studied topic in the field. Much work has been done to understand the Hsp90 chaperone cycle, the effects of cochaperones, and cochaperone stoichiometry in protein complexes. Many Hsp90 complexes in the absence of clients exist in symmetric configurations with both protomers populating similar conformations [30,130,131,132,133]. Though, an asymmetric conformation of the mitochondrial Hsp90 in absence of client has been observed [114]. Hsp90s also exist in asymmetric states that are important for client protein folding. A combination of biochemical and biophysical experiments have suggested that Hsp90 interacts asymmetrically with client proteins [134]. One study has shown that Hsp90 can bind two client proteins simultaneously [135], but cochaperones such as Cpr6 or p23 change the stoichiometry of GR binding and induce asymmetry in the chaperone cycle [22]. The current model of protein remodeling in bacterial systems begins with the DnaK chaperone system, including a J protein and NEF, in client recognition and early remodeling. The client will then be transferred from DnaK to Hsp90, through the direct interaction of the chaperones, for subsequent remodeling [72]. The asymmetric model presented in this work is consistent with this mechanism. If only one client protein can be acted upon by Hsp90, then only one DnaK molecule would be needed to transfer a client.

While the work in this study is focused on the interactions between bacterial Hsp90 and Hsp70, the insights gained here may be valuable for further examinations of eukaryotic Hsp90 and Hsp70 family members. Hsp90${}_{Ec}$ and DnaK are about 40–50% similar to mammalian homologs; they have been shown to be highly similar in structure and function [31,136]. The results from SPM and sequence alignment show that the most important residues for allosteric conformational changes in these chaperones are highly conserved throughout the Hsp90 and Hsp70 families. Unlike the bacterial systems, eukaryotic Hsp90 has at least 20 cochaperones that participate in targeting client proteins to Hsp90 and modulate the conformational dynamics in the Hsp90 protein remodeling cycle [9,39,75,76,137,138,139,140,141,142,143,144]. These eukaryotic cochaperones may interact symmetrically or asymmetrically at different points during the chaperone cycle. The Hop/Sti1 cochaperone inhibits the ATPase activity of Hsp90 and facilitates the interaction between Hsp70 and Hsp90. In complexes with only eukaryotic Hsp90 and Hop/Sti1, varying symmetry has been observed with stoichiometry of 1:1 or 2:1, respectively [77,145,146]. However, one Hop/Sti1 molecule effectively stabilized Hsp90 and rendered it incompetent for ATP hydrolysis [77,147,148]. Similarly, the cochaperone p23/Sba1 is also an inhibitor of ATPase activity, acting late in the chaperone cycle to stabilize the closed conformation of Hsp90. The Hsp90-p23 complex was resolved in a symmetric conformation [116], though biochemical experiments observed asymmetric binding in vitro [149,150]. Other cochaperones such as Aha1, a stimulator of ATPase activity, bind to one protomer and facilitate ATP hydrolysis of both Hsp90 protomers [151].

Asymmetry is common in ternary Hsp90 complexes, similar to the observation of binary complexes with cochaperones. The cochaperone Cdc37 facilitates the delivery of client kinases to eukaryotic Hsp90 [152], similar to the role of DnaK. Ternary complexes of a client protein, Cdk4, with Cdc37 and Hsp90 indicate asymmetry in the complex with one Cdc37 and Cdk4 per Hsp90 dimer [18,153]. Another cochaperone, Hop/Sti1, facilitates the interaction between Hsp70 and Hsp90 [39], though a direct interaction between Hsp70 and Hsp90 in the absence of this cochaperone has been observed [67]. Multiple structures of the client loading complex of eukaryotic Hsp90s with Hsp70, Hop/Sti1 and a model client protein, glucocortocoid receptor (GR) have been resolved. While the exact binding configurations differ and may provide snapshots in time of the dynamics in remodeling mechanisms, the stoichiometry involves asymmetry in Hop/Sti1 and Hsp70 binding to the Hsp90 dimer [14,146]. During preparation of this manuscript, a recent cryo-EM structure of this client loading complex in high resolution was made available. In contrast to previous observations, this complex was symmetric in Hsp70 binding with one molecule bound at each Hsp90 protomer within the homologous region indicated in Figure 1 from the *E. coli* system. However, the two Hsp70 SBDs are not fully visible. In the low resolution structures, one Hsp70 SBD can be visualized interacting at the M-C domain of an Hsp90 protomer while the second Hsp70 SBD is still not resolved. This could be due to the inherent flexibility of the DnaK SBD and DnaK not populating a stable conformation, such as the ADP bound state as observed in the other DnaK molecule in this complex.

In this work we provide evidence to support asymmetric binding of DnaK by Hsp90. This system in bacteria does not include a homologous Hop/Sti1 bridging protein to facilitate the transfer of a client; hence, DnaK may be playing the role of this cochaperone in *E. coli* to arrest Hsp90 in a stable conformation for client transfer. In symmetric DnaK bound complexes, we have only investigated models that include two DnaK in the ADP bound conformation. We cannot rule out additional asymmetric complexes where DnaK populates an intermediate state. The current structures of DnaK available include those used in this study in the ADP bound state and the ATP bound conformation [11,50]. In previous studies it has been shown that the ATP bound conformation is incompatible with Hsp90${}_{Ec}$ binding, because the same interface on the DnaK NBD that interacts with Hsp90${}_{Ec}$ also interacts with the DnaK SBD. To our knowledge, no undocked NBD-SBD DnaK conformations exist that render the Hsp90${}_{Ec}$ interacting region of DnaK available for binding. Future studies of DnaK conformations are needed to explore this avenue. Overall, this work is beginning to shed light on the molecular details in the coupling of the Hsp90 and DnaK chaperone cycles and how the chaperones modulate each other.

## Data Availability

The data presented in this study are available upon request from the corresponding authors.

## Supplementary Materials

The following are available online at https://www.mdpi.com/1422-0067/22/4/2200/s1, Figure S1: Residue fluctuations in the dominant normal modes of Hsp90${}_{Ec}$ alone, Figure S2: Residue fluctuations in the dominant normal modes of asymmetric DnaK bound Hsp90${}_{Ec}$, Figure S3: Residue fluctuations in the dominant normal modes of symmetric DnaK bound Hsp90${}_{Ec}$, Figure S4: Hot-spot residues for the symmetric Hsp90${}_{Ec}$-DnaK models, Figure S5: Alignment of DnaK and 10 other homologous Hsp70 family members, Figure S6: Amino acid sequence alignment of Hsp90${}_{Ec}$ and 7 other homologous Hsp90 family members, Table S1: Hot-spot residues predicted by SPM analysis for each mode of each model, Video S1: Hsp90${}_{Ec}$ alone Mode 7, Video S2: Hsp90${}_{Ec}$ alone Mode 7 as viewed at a 90${}^{\circ }$ angle to Video S1, Video S3: Hsp90${}_{Ec}$ alone Mode 8, Video S4: Hsp90${}_{Ec}$ alone Mode 8 as viewed at a 90${}^{\circ }$ angle to Video S3, Video S5: Hsp90${}_{Ec}$-1DnaK Mode 8, Video S6: Hsp90${}_{Ec}$-1DnaK Mode 8 as viewed at a 90${}^{\circ }$ angle to Video S5, Video S7: Hsp90${}_{Ec}$-1DnaK Mode 10, Video S8: Hsp90${}_{Ec}$-1DnaK Mode 10 as viewed at a 90${}^{\circ }$ angle to Video S7, Video S9: Hsp90${}_{Ec}$-2DnaK Mode 13, Video S10: Hsp90${}_{Ec}$-1DnaK Mode 13 as viewed at a 90${}^{\circ }$ angle to Video S9, Video S11: Hsp90${}_{Ec}$-2DnaK Mode 28, Video S12: Hsp90${}_{Ec}$-2DnaK Mode 28 as viewed at a 90${}^{\circ }$ angle to Video S11, Video S13: Hsp90${}_{Ec}$-2DnaK Mode 8, Video S14: Hsp90${}_{Ec}$-2DnaK Mode 8 as viewed at a 90${}^{\circ }$ angle to Video S13.

## Abbreviations

The following abbreviations are used in this manuscript:

SBD	substrate binding domain
NBD	nucleotide binding domain
HtpG	Hsp90${}_{Ec}$
DnaK	Hsp70
client	substrate
NTD	N-terminal domain
MD	middle domain
CTD	C-terminal domain
ENM	Elastic Network Model
NMA	Normal Mode Analysis
SPM	Structural Perturbation Method
