# Supplementary figures and images for: Structural Communication between the E. coli Chaperones DnaK and Hsp90
Source: Int J Mol Sci. 2021 Feb 23;22(4):2200. doi: 10.3390/ijms22042200 (PMC7926864; doi:10.3390/ijms22042200)

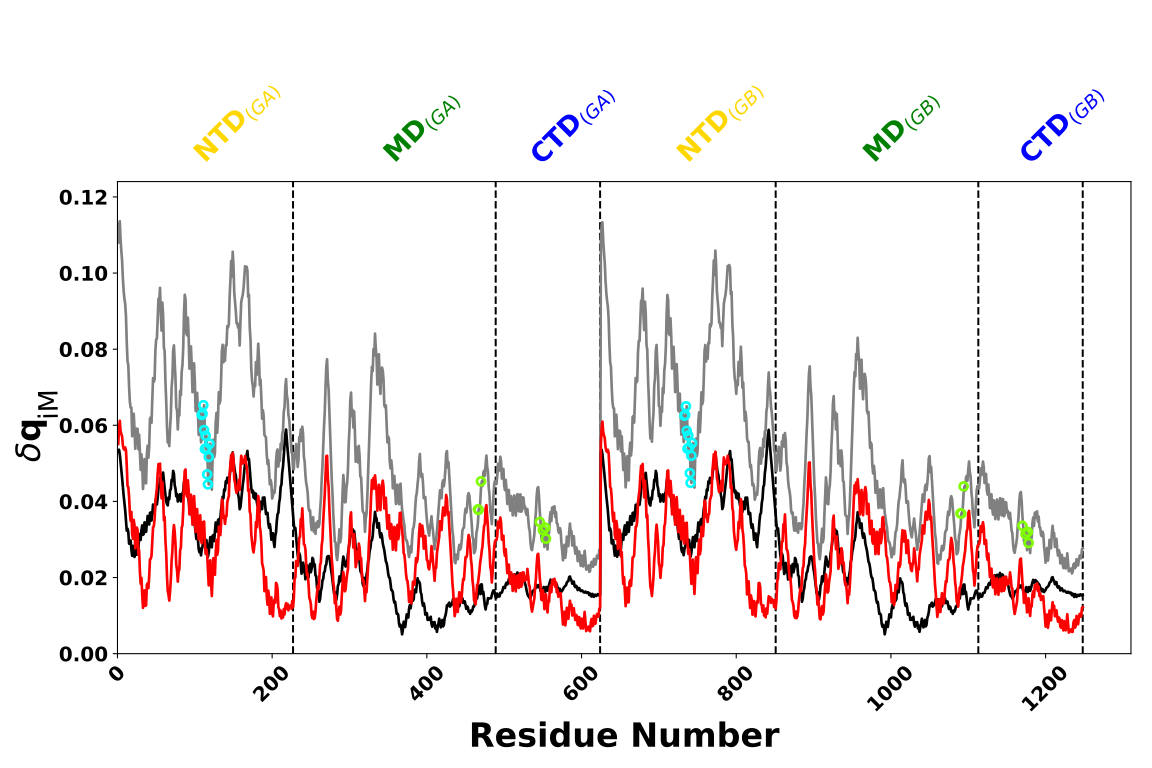

Supplement: Supplementary file 1 [file ijms-22-02200-s001.zip › figs1.png]

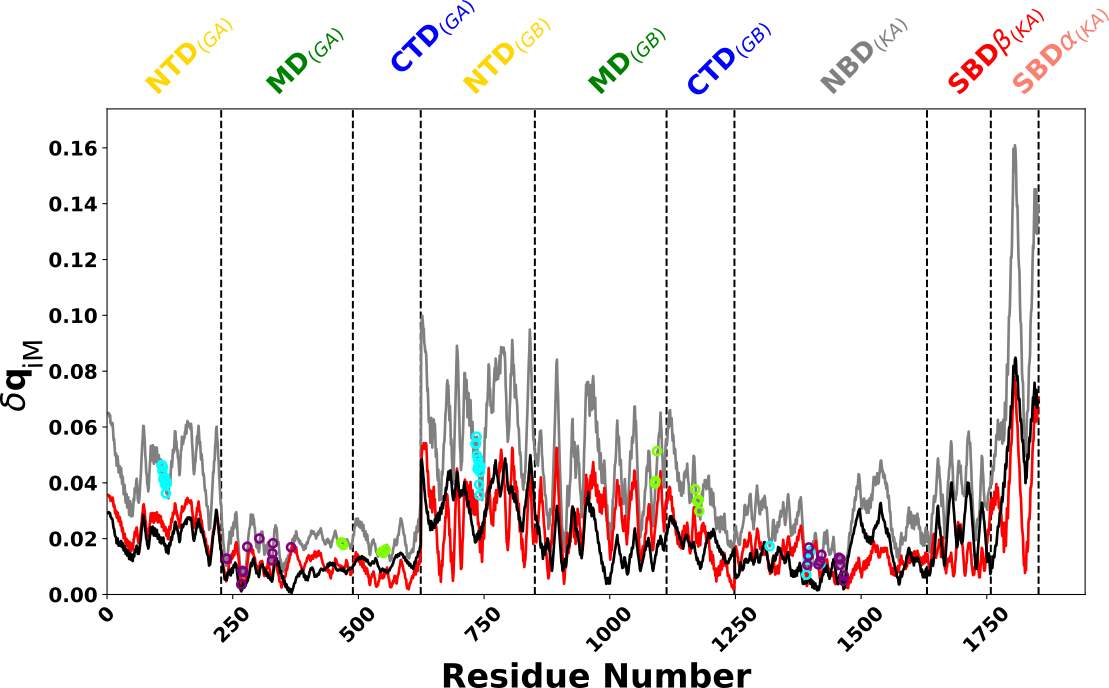

Supplement: Supplementary file 1 [file ijms-22-02200-s001.zip › figs2.png]

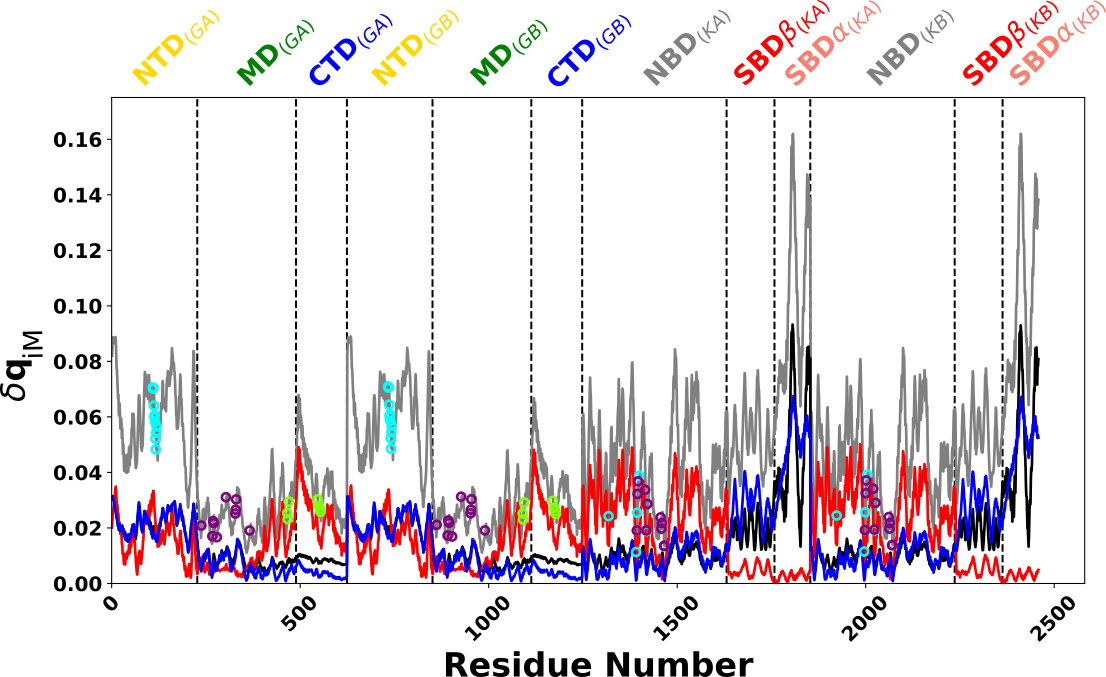

Supplement: Supplementary file 1 [file ijms-22-02200-s001.zip › figs3.png]

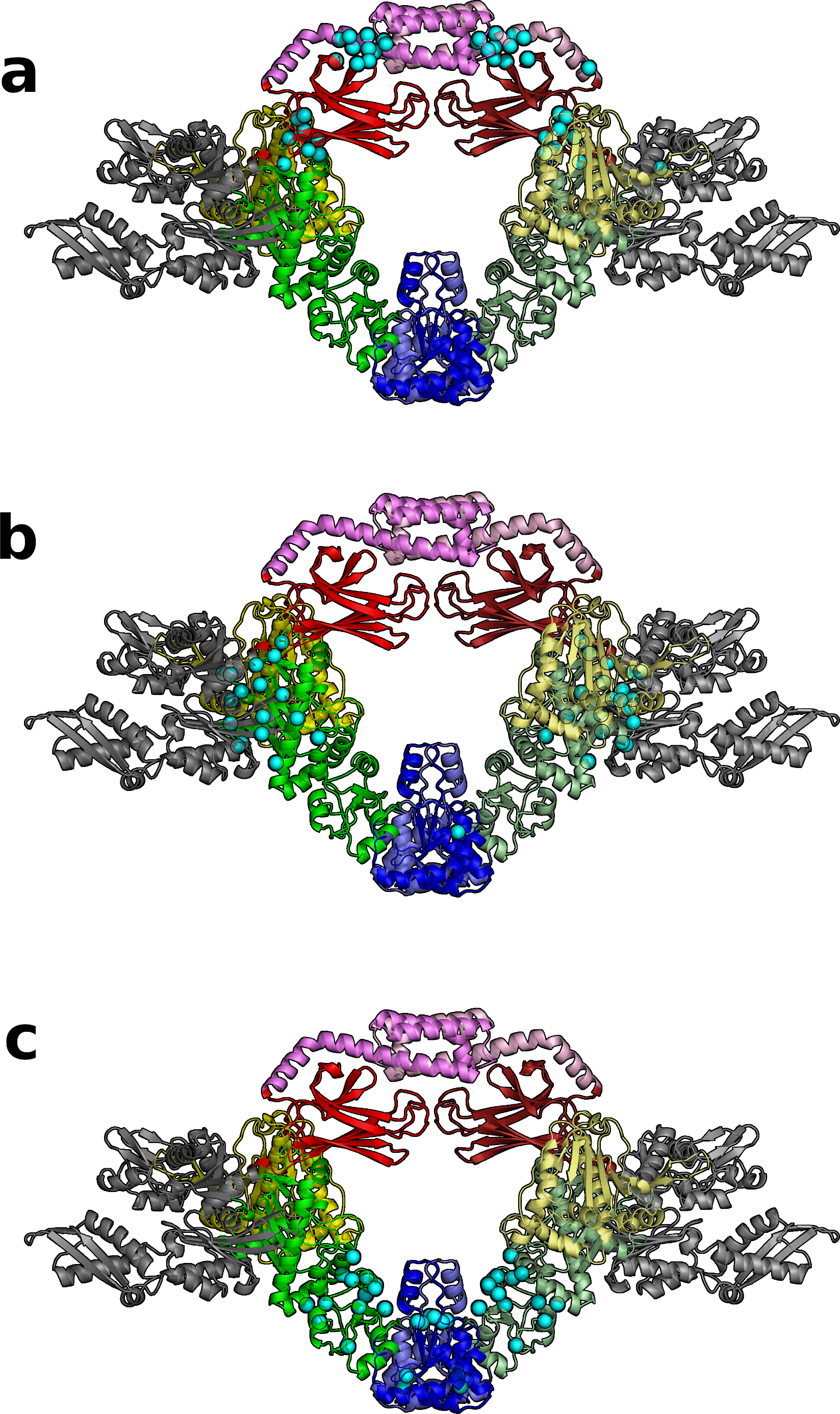

Supplement: Supplementary file 1 [file ijms-22-02200-s001.zip › figs4.png]

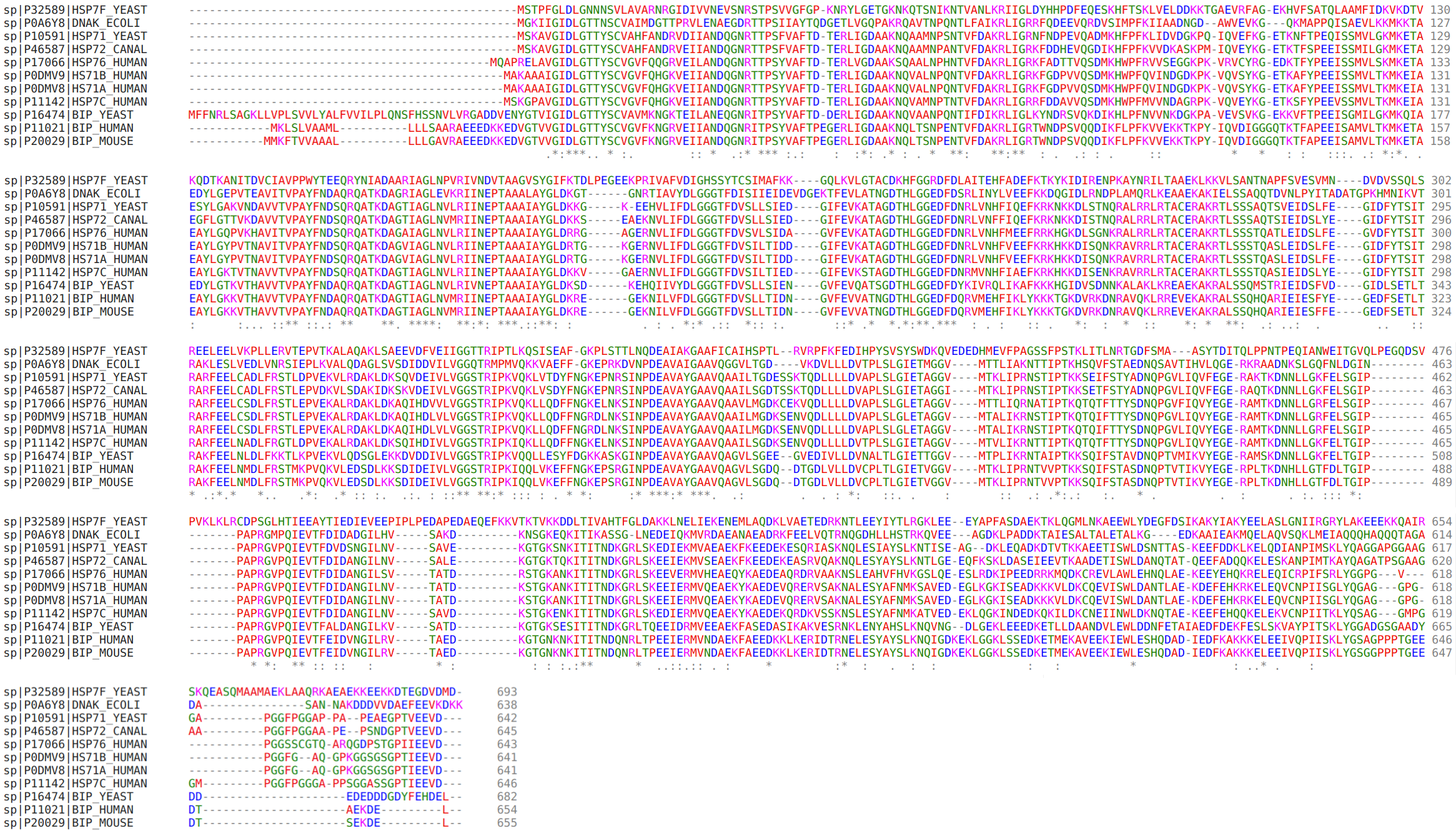

Supplement: Supplementary file 1 [file ijms-22-02200-s001.zip › figs5.png]

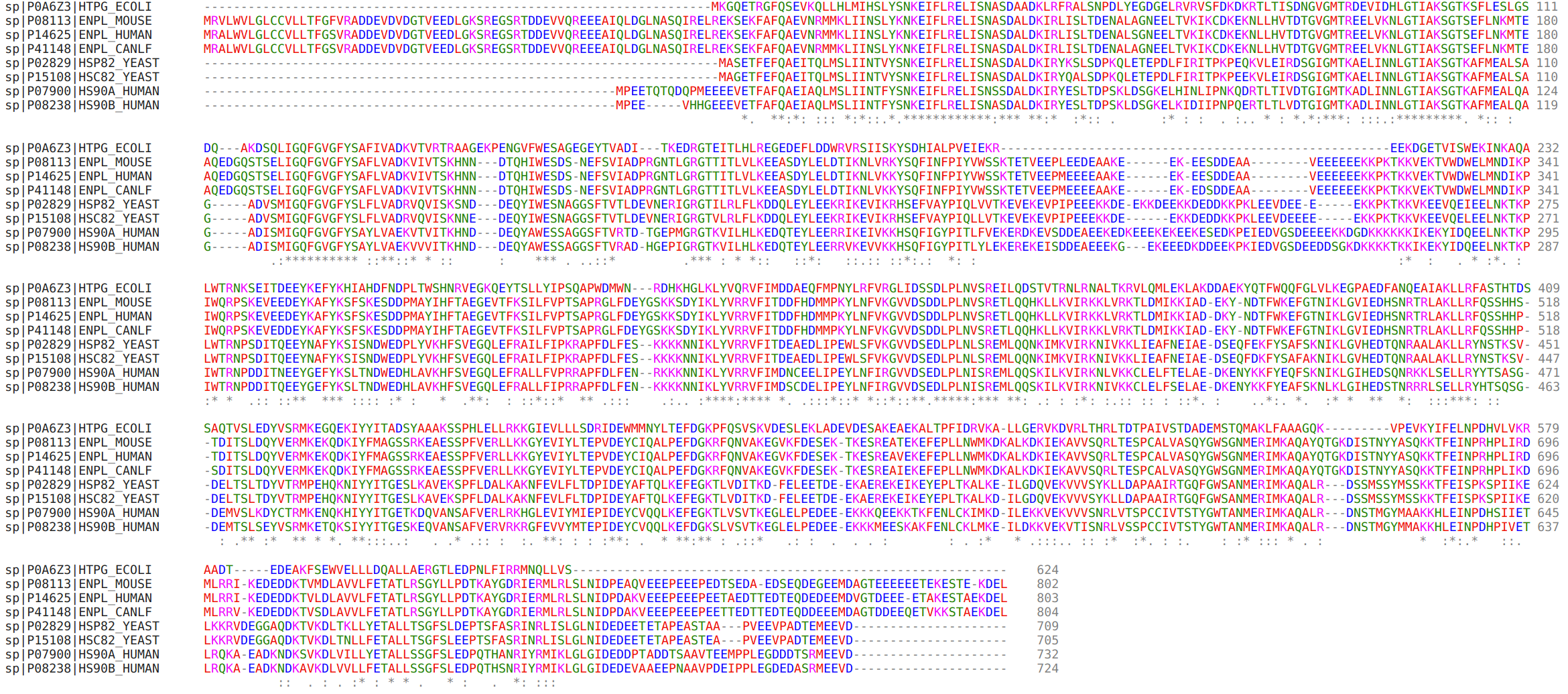

Supplement: Supplementary file 1 [file ijms-22-02200-s001.zip › figs6.png]

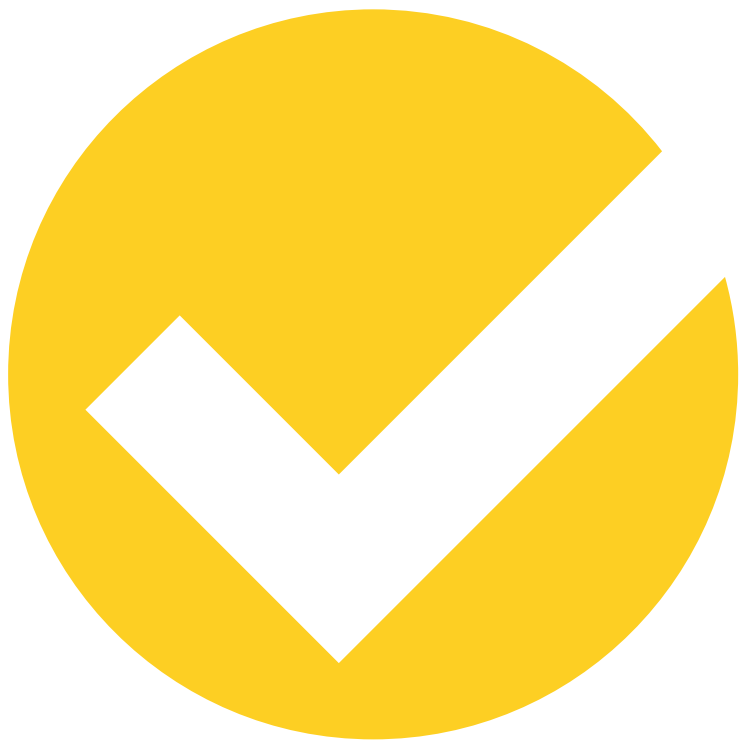

check for  
updates

Supplement: Supplementary file 1 [file ijms-22-02200-s001.zip › Definitions/logo-updates.pdf]
